# Prediction of Pregnancy-Associated Hypertension Using a Scoring System: A Multicenter Cohort Study

**DOI:** 10.3390/life13061330

**Published:** 2023-06-06

**Authors:** Yun Sung Jo, Woo Jeng Kim, Sae Kyung Choi, Su Mi Kim, Jae Eun Shin, Ki Cheol Kil, Yeon Hee Kim, Jeong Ha Wie, Han Wool Kim, Subeen Hong, Hyun Sun Ko

**Affiliations:** 1 Department of Obstetrics and Gynecology, St. Vincent’s Hospital, College of Medicine, The Catholic University of Korea, Seoul 06591, Republic of Korea; eggs76@catholic.ac.kr; 2 Department of Obstetrics and Gynecology, Incheon St. Mary’s Hospital, College of Medicine, The Catholic University of Korea, Seoul 06591, Republic of Korea; yoteamo@catholic.ac.kr (W.J.K.); obgysk@catholic.ac.kr (S.K.C.); 3 Department of Obstetrics and Gynecology, Daejeon St. Mary’s Hospital, College of Medicine, The Catholic University of Korea, Seoul 06591, Republic of Korea; alex4yu@catholic.ac.kr; 4 Department of Obstetrics and Gynecology, Bucheon St. Mary’s Hospital, College of Medicine, The Catholic University of Korea, Seoul 06591, Republic of Korea; jennie1008@catholic.ac.kr; 5 Department of Obstetrics and Gynecology, Yeouido St. Mary’s Hospital, College of Medicine, The Catholic University of Korea, Seoul 06591, Republic of Korea; kilssine@catholic.ac.kr; 6 Department of Obstetrics and Gynecology, Uijeongbu St. Mary’s Hospital, College of Medicine, The Catholic University of Korea, Seoul 06591, Republic of Korea; yoni@catholic.ac.kr; 7 Department of Obstetrics and Gynecology, Eunpyeong St. Mary’s Hospital, College of Medicine, The Catholic University of Korea, Seoul 06591, Republic of Korea; wiejh@catholic.ac.kr; 8 Department of Obstetrics and Gynecology, Seoul St. Mary’s Hospital, College of Medicine, The Catholic University of Korea, Seoul 06591, Republic of Korea; wool829@cmcnu.or.kr (H.W.K.); unihsy@catholic.ac.kr (S.H.)

**Keywords:** pregnancy-associated hypertension, prediction, risk, scoring

## Abstract

This study aimed to develop an early pregnancy risk scoring model for pregnancy-associated hypertension (PAH) based on maternal pre-pregnancy characteristics, such as mean arterial pressure (MAP), pregnancy-associated plasma protein-A (PAPP-A) or neither. The perinatal databases of seven hospitals from January 2009 to December 2020 were randomly divided into a training set and a test set at a ratio of 70:30. The data of a total pregnant restricted population (women not taking aspirin during pregnancy) were analyzed separately. Three models (model 1, pre-pregnancy factors only; model 2, adding MAP; model 3, adding MAP and PAPP-A) and the American College of Obstetricians and Gynecologists (ACOG) risk factors model were compared. A total of 2840 (8.11%) and 1550 (3.3%) women subsequently developed PAH and preterm PAH, respectively. Performances of models 2 and 3 with areas under the curve (AUC) over 0.82 in both total population and restricted population were superior to those of model 1 (with AUCs of 0.75 and 0.748, respectively) and the ACOG risk model (with AUCs of 0.66 and 0.66) for predicting PAH and preterm PAH. The final scoring system with model 2 for predicting PAH and preterm PAH showed moderate to good performance (AUCs of 0.78 and 0.79, respectively) in the test set. “A risk scoring model for PAH and preterm PAH with pre-pregnancy factors and MAP showed moderate to high performances. Further prospective studies for validating this scoring model with biomarkers and uterine artery Doppler or without them might be required”.

## 1. Introduction

Pregnancy-associated hypertension (PAH) affects about 5.0–13.0% in the world [1]. It is one of the major obstetric complications associated with maternal and fetal mortality and morbidity [2]. Several studies have shown that low-dose aspirin administration can help prevent preeclampsia (PE), which is the most dangerous form of hypertensive disorders in pregnancy (HDP) [3,4,5]. Thus, low-dose aspirin is recommended for the high-risk groups of PE [6,7,8]. However, the criteria of the high-risk groups for low-dose aspirin use vary slightly from country to country (United States: The American College of Obstetrician and Gynecologists (ACOG) guideline [6], United Kingdom: National Institute for Health and Excellence’s (NICE) guideline [7]). In addition, the proportion of Asian origin women in most studies about PE has been very small (less than 10%) [8,9]. In 2019, the Fetal Medicine Foundation (FMF) suggested a new predictive model with a high predictive rate of gestational hypertension by combining mean arterial pressure (MAP), uterine artery Doppler, and biochemical markers with some characteristics of the mothers [10]. The model suggested by the FMF contained some basic characteristics of the mothers. However, a systematic literature review has revealed that when basic characteristics of the mothers are fully utilized, the predictive power is excellent [11]. In addition, because race, healthcare system, and socioeconomic conditions are all different, a predictive model should be based on basic characteristics first. Studies should also determine whether the predictive power can be improved when other factors, such as biomarkers and ultrasound parameters, are added while considering cost effectiveness. As the importance of planned pregnancy and periconceptional counseling is emphasized [12], efforts should be made accordingly. Recently, high-risk pregnancy accompanied by chronic disease is increasing due to an increase in maternal age [13,14]. Therefore, predicting the high-risk group for PAH and prophylaxis is important.

Although it is challenging to compare the results of PAH prediction with the previous findings of PE prediction [8,9,10,11], this study aimed to develop a preliminary predication model for PAH in Korea, taking into account the PE risk factors outlined in global guidelines. MAP and Pregnancy-associated plasma protein-A (PAPP-A), one of the routine first trimester screening markers for aneuploidy, were evaluated as risk factors. The purpose of this study was to develop a preliminary early pregnancy risk scoring model for PAH according to basic characteristics of mothers before pregnancy with MAP and PAPP-A or without them.

## 2. Materials and Methods

### 2.1. Data Source

This was a retrospective study using the electronic medical record (EMR) systems of seven secondary or tertiary center hospitals under the College of Medicine, the Catholic University of Korea, from January 2009 to December 2020. Among pregnant women who delivered at the seven hospitals during the period, those with maternal age less than 18 years were excluded from analysis. Basic prenatal characteristics (maternal, social, reproductive, medical, and family history) and clinical characteristics (blood pressure, height, and weight) were retrieved from the EMR. This study was approved by the Institutional Review Board (XC20WIDI0103/2020-2158-0020) at each of the seven hospitals. Informed consent was waived due to its retrospective nature.

### 2.2. Study Design

PAH was defined as gestational hypertension, PE, eclampsia, or chronic hypertension with superimposed PE [1,15]. International Classification of Diseases-10 (ICD10) codes (O11: superimposed PE, O13: gestational hypertension, O14: PE, O15: eclampsia, and O16: unspecified maternal hypertension) were used to extract the PAH group from the EMR. The criteria of gestational hypertension are new-onset hypertension, indicated by systolic and diastolic blood pressures exceeding 140 mmHg and 90 mmHg, respectively, after 20 weeks’ gestation [15]. PE is characterized by hypertension accompanied by proteinuria or multiorgan involvement, which may manifest as thrombocytopenia, renal dysfunction, hepatocellular necrosis, central nervous system perturbations, or pulmonary edema [15]. The criteria of PE were hypertension plus proteinuria or multiorgan involvement reflected by thrombocytopenia, renal dysfunction, hepatocellular necrosis, central nervous system perturbations, or pulmonary edema [15]. Superimposed PE is diagnosed in women who have PE superimposed on chronic hypertension. Eclampsia is diagnosed in women with PE and new-onset seizures in the absence of other causative conditions [15]. Unspecified maternal hypertension refers to the diagnosis of hypertension in women within 40 weeks before or 12 weeks after delivery who do not meet the criteria for either chronic or pregnancy-induced hypertension [16].

To confirm the diagnosis and fill in missing data, a chart review was done by two maternal fetal medicine doctors (H.S.K. and J.H.W.). Preterm PAH was defined as cases who had a delivery before 37 weeks of gestation due to PAH. Baseline and clinical characteristics of the group affected by PAH and the group without PAH (control) were compared. Prepregnant maternal body mass index (BMI, kg/m^2^) before pregnancy was calculated from measured height, and weight (at delivery), and self-reported prepregnant body weight. Maternal blood pressure was measured on the right upper arm using manual blood pressure equipment with a cuff size appropriate for arm circumference. Korotkoff V was used for diastolic blood pressure.

The history of previous pregnancy complications, including PE, fetal death in utero (FDIU), fetal growth restriction (FGR), gestational diabetes (GDM), and preterm birth before 37 weeks of gestation, was obtained from the obstetric record. The diagnosis of GDM in the current pregnancy was based on the ICD code assigned during pregnancy (ICD10 codes O24.4 or O24.9), excluding women with a pre-pregnancy record of diabetes (defined as any of the ICD10 codes O24.0–24.3 or E12–14) or prescription codes for insulin or other diabetes medication. GDM screening test results were also considered using a two-step procedure routinely performed for all women at 24–28 weeks of pregnancy, following NIH guidelines [17,18]. The first recorded blood pressure before 20 weeks of gestation was collected for women with available data. MAP, calculated as (systolic blood pressure + (2 × diastolic blood pressure))/3, was used in prediction models (models 2 and 3). Additionally, multiples of the median (MoM) values of PAPP-A in maternal serum screening tests during the first trimester were extracted for women with available data and used in model 3. Maternal serum PAPP-A was measured between 11 + 0 and 13 + 6 weeks of pregnancy as part of a routine aneuploidy screening. To account for the variations in serum marker concentrations according to gestational age, MoM values of PAPP-A for the corresponding gestational age were retrieved from the screening records for women with available data.

### 2.3. Restricted Population

To avoid the influence of aspirin during pregnancy, another analysis for the restricted population after excluding women who took aspirin during pregnancy based on the medical records was performed.

### 2.4. Datasets of Original and Restricted Populations

The original dataset was randomly divided into two sub-cohorts (a model development cohort or training set) and a validation cohort or test set) at a ratio of 70:30. The data set of the restricted population was also divided into training and test sets at a ratio of 70:30.

### 2.5. Statistics

All the analyses were performed using SAS software, version 9.4 (SAS Institute, Inc., Cary, NC, USA). A chi-squared test and a two-sample t-test were used for comparing the variables of the study population for women who did or did not develop PAH. Bivariate associations of PAH and each predictor variable were evaluated with a chi-square test for categorical variables and a t-test for continuous variables. Variables with a statistical difference in the univariate analysis were included in the multivariable stepwise logistic regression models. After comparing the performances of the three different multivariable regression models (model 1, clinical factor only; model 2, clinical factor and MAP; and model 3, clinical factor, MAP, and PAPP-A) based on the values of the area under the curve (AUC) and the sensitivity of PAH at 10% false positive rate (FPR), a statistic scoring model was developed to stratify the risks for PAH. The performance of the ACOG risk classification guideline for the prevention of PE was also evaluated. Diagnostic accuracy of the scoring system was performed with receiver operating characteristic (ROC) curves. Statistical significance was defined as a two-sided *p*-value < 0.05.

## 3. Results

### 3.1. Baseline Characteristics

**Figure 1:**
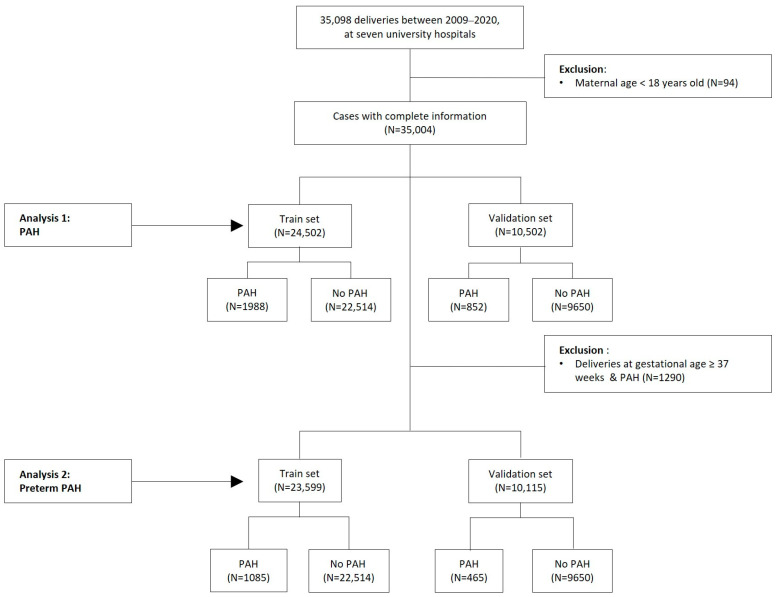
Participants flow chart of total population. PAH, Pregnancy-associated hypertension.

**Figure A1:**
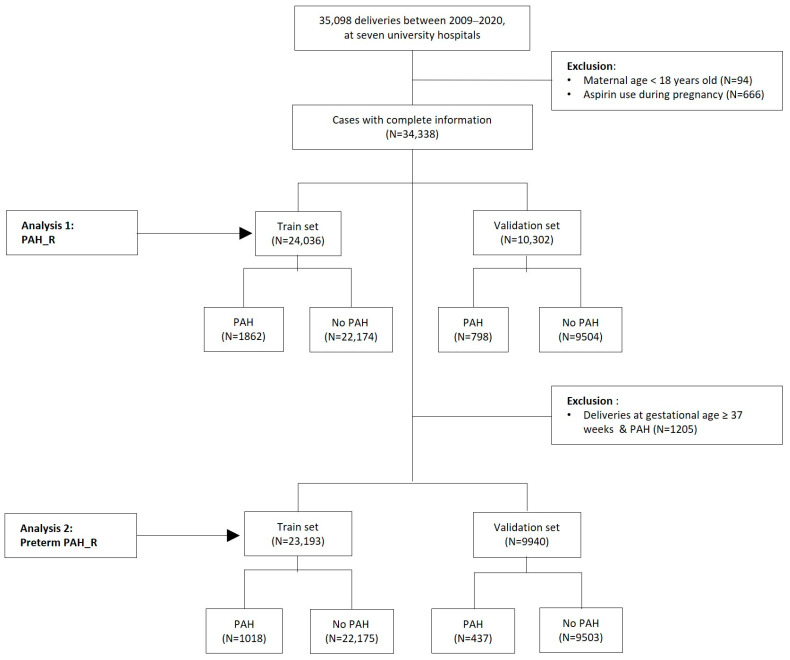
Participants flow chart of restricted population. PAH, Pregnancy-associated hypertension; PAH_R, Pregnancy-associated hypertension of restricted study population without aspirin treatment.

In a total of 35,098 cases of delivery, 35,004 cases were included, excluding 94 cases with an age less than 18 years. There were a total of 2840 pregnant women with PAH (1988 women in the training set and 852 women in the validation set), with an incidence rate of 8.11%. There were a total of 1550 pregnant women with preterm PAH (1085 women in the training set and 465 women in the validation set), with an incidence rate of 3.30% (Figure 1). A flow chart for the restricted population after excluding 666 women who took aspirin during pregnancy is presented in Figure A1.

Baseline and clinical characteristics of the subjects in the training set for analysis 1 and analysis 2 are presented in Table 1. The mean maternal age, BMI before pregnancy, proportions of nulliparity, pregnancies by in vitro fertilization, multiple pregnancy, family history of hypertension, history of hypertension, renal disease, hyperlipidemia, diabetes, insulin glucose tolerance (IGT), lupus or anti-phospholipid syndrome (APS), other rheumatic diseases, and aplastic anemia were significantly higher in the PAH group and the preterm PAH group than in the control group. Pregnancies with a smoking and drinking history and a history of IGT were significantly higher in the PAH group, but not significantly higher in the preterm PAH group, compared to the control group. In multiparous women, histories of PE, FDIU, FGR, GDM, and preterm birth in a previous pregnancy were significantly higher in both PAH and preterm PAH groups than in the control group. The MAPs before 20 weeks of gestation were available for 4822 women. There were significant differences in the MAP between the PAH and control groups (95.4 ± 14.44 mmHg vs. 83.36 ± 9.73 mmHg, *p* < 0.001) and between the preterm PAH and control groups (100.21 ± 17.44 mmHg vs. 83.36 ± 9.73 mmHg, *p* < 0.0001). MoM values of PAPP-A between 11 weeks and 13 weeks of gestation were available for 4748 women. There were significant differences in the MoM value between the PAH and control groups (1.04 ± 0.69 MoM vs. 1.19 ± 0.66 MoM, *p* < 0.0001) and between the preterm PAH and control groups (1.04 ± 0.71 MoM vs. 1.19 ± 0.66 MoM, *p* = 0.0044). The baseline and clinical characteristics of subjects in the training set of the restricted population are presented in Table A1.

### 3.2. Identifying Risk Factors Using Univariate and Multivariate Logistic Regression Analyses

#### 3.2.1. Univariate and Multivariate Logistic Regression Analyses of Risk Factors for PAH

- A preliminary study in a population of patients from Korea

Using significant variables between the PAH and control groups in a univariate analysis, a stepwise multivariate logistic regression analysis was performed. Significant variables in the multivariate analysis with maternal clinical factors only (model 1) were multiple pregnancy (OR: 1.515, 95% CI: 1.267–1.813), maternal age (OR: 1.023, 95% CI: 1.011–1.034), nulliparity (OR: 2.017, 95% CI: 1.809–2.249), history of drinking (OR: 2.019, 95% CI: 1.222–3.334), previous history of PE (OR: 3.654, 95% CI: 2.843–4.697), BMI before pregnancy (OR: 1.143, 95% CI: 1.131–1.155), family history of hypertension (OR: 1.645, 95% CI: 1.476–1.834), past history of hypertension (OR: 4.410, 95% CI: 3.748–5.188), diabetes (OR: 1.572, 95% CI: 1.119–2.208), renal disease (OR: 2.137, 95% CI: 1.542–2.961), and lupus or APS (OR: 1.722, 95% CI: 1.154–2.571) (Table 2). When another analysis for model 2 was performed for women with MAPs before 20 weeks of gestation, MAP was significantly associated with PAH (OR: 1.066, 95% CI: 1.054–1.079) (Table 3). When the last analysis for model 3 was performed for women with MAPs before 20 weeks of gestation and PAPP-A values, the MAPs and MoM values of PAPP-A were significantly associated with PAH (OR: 1.055, 95% CI: 1.036–1.075 and OR: 0.473, 95% CI: 0.265–0.844, respectively) (Table A2).

#### 3.2.2. Univariate and Multivariate Logistic Regression Analyses of Risk Factors for Preterm PAH-Additional Analysis

Using significant variables between the PAH and control groups in a univariate analysis, a stepwise multivariate logistic regression analysis was performed. In the multivariate analysis with maternal clinical factors only (model 1), the following variables were significant: multiple pregnancy (OR: 2.328, 95% CI: 1.901–2.851), maternal age (OR: 1.017, 95% CI: 1.002–1.032), nulliparity (OR: 1.749, 95% CI: 1.519–2.013), previous history of PE (OR: 3.275, 95% CI: 2.385–4.498), BMI before pregnancy (OR: 1.137, 95% CI: 1.122–1.153), family history of hypertension (OR: 1.536, 95% CI: 1.334–1.769), past history of hypertension (OR: 3.937, 95% CI: 3.186–4.865), diabetes (OR: 1.625, 95% CI: 1.049–2.518), renal disease (OR: 2.744, 95% CI: 1.855–4.058), and lupus or APS (OR: 2.108, 95% CI: 1.311–3.389) (Table 4). When another analysis for model 2 was performed for women with MAPs before 20 weeks of gestation, MAP was significantly associated with PAH (OR: 1.083, 95% CI: 1.067–1.100) (Table 5). When the last analysis for model 3 was performed for women with MAPs before 20 weeks of gestation and PAPP-A values, MAPs and MoM values of PAPP-A were significantly associated with PAH (OR: 1.081, 95% CI: 1.056–1.10 and OR: 0.417, 95% CI: 0.181–0.959, respectively) (Table A3).

#### 3.2.3. Performance of Scoring Models for Predicting PAH and Preterm PAH in Total and Restricted Populations

AUC values for predicting PAH and preterm PAH in the total and restricted populations without aspirin treatment during pregnancy are given in Table 6. Based on the variables included, AUCs in models 2 and 3 reached over 0.82 in both total and restricted populations, indicating a moderate-to-high predictive ability. Sensitivities at a 10% FPR for predicting PAH and preterm PAH were higher with model 2 (54.8% and 66.4%, respectively) and model 3 (53.8% and 60.3%, respectively) than with model 1 (41.0% and 38.9%, respectively) for the total population. Sensitivities at a FPR of 10% for predicting PAH and preterm PAH were higher with model 2 (52.2% and 65.5%, respectively) and model 3 (50.4% and 58.1%, respectively) than with model 1 (41% and 41.5%, respectively) for the restricted population. When risk factors of the ACOG risk classification guidelines for preventing PE were applied, AUC was 0.67 in the total population and 0.66 in the restricted population. Sensitivities at a FPR of 10% were 31.0% and 29.4% in the total population and the restricted population, respectively.

#### 3.2.4. Development of a Scoring System with Validation

**Figure 2:**
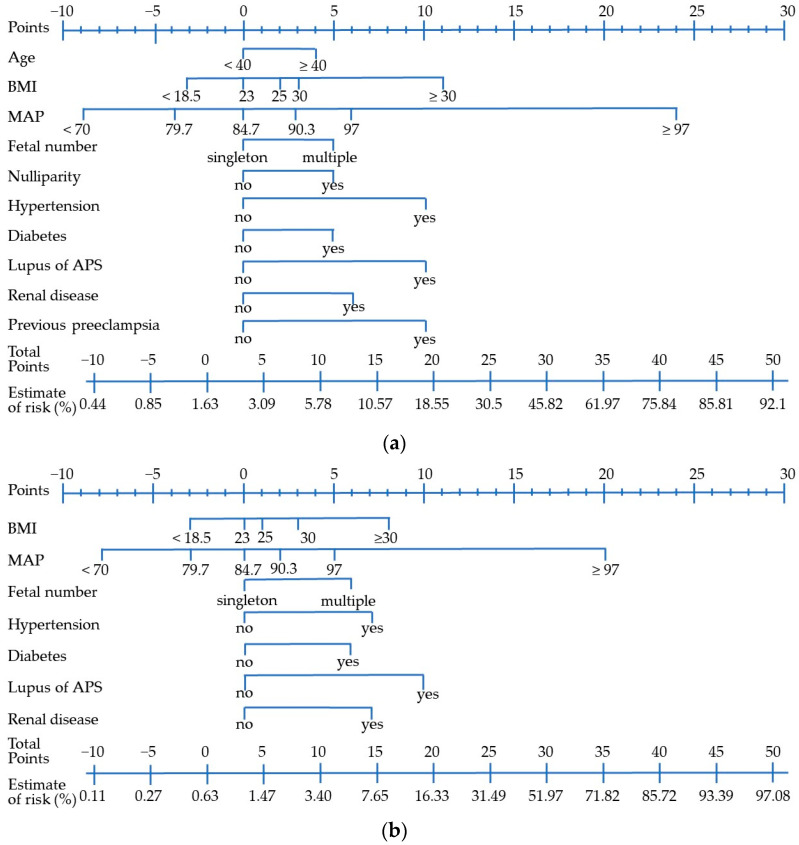
Scoring system: (**a**) The scoring system based on 10 early pregnancy risk factors for pregnancy-associated hypertension. The total score (ranging −12 to 85) is calculated by summing the individual scores for each risk factor; (**b**) The scoring system based on 7 early pregnancy risk factors for preterm pregnancy-associated hypertension. The total score (ranging −12 to 75) is calculated by summing the individual scores for each risk factor. APS: antiphospholipid syndrome; BMI: Body mass index; MAP: Mean arterial pressure.

**Figure 3:**
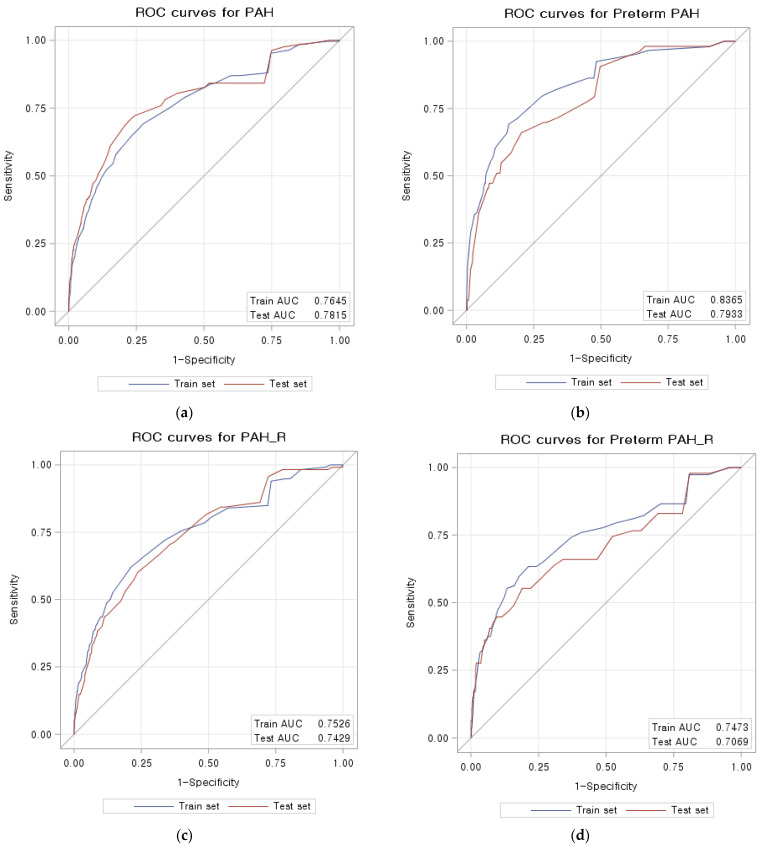
ROC curves: (**a**) PAH of total population; (**b**) preterm PAH of total population; (**c**) PAH of restricted population; (**d**) preterm PAH of restricted population. PAH, pregnancy-associated hypertension.

Based on performance results, the scoring systems for PAH and preterm PAH were constructed using model 2 (Figure 2a,b). BMI and MAP were divided into 5 and 6 categories, respectively. Other factors (age, nulliparity, fetal number in this pregnancy, history of hypertension, diabetes, lupus or APS, renal disease, and history of previous preeclampsia) were divided into two categories. The ROC curves of the scoring system with the training set and the test set to predict PAH (AUC = 0.76 in the training set and AUC = 0.78 in the test set) and preterm PAH (AUC = 0.84 in the training set and AUC = 0.79 in the test set) are given in Figure 3. In the restricted population, the ROC curves of the scoring system showed similar results for predicting PAH (AUC = 0.75 in the training set and AUC = 0.74 in the test set) and preterm PAH (AUC = 0.75 in the training set and AUC = 0.71 in the test set).

## 4. Discussion

In this study, the incidences of PAH and preterm PAH were 8.11% and 3.30%, respectively, which were similar to the recent prevalence of HDP in Korea (8.0–9.0%) [16]. However, the cases with chronic hypertension only were not included in the outcomes of PAH, as there is no need to predict it unless superimposed PE develops. The incidence of PAH was relatively high compared to the known incidence [1]. This might be attributed to the fact that secondary or tertiary hospitals have a higher proportion of high-risk pregnancies.

This study investigated independent early pregnancy risk factors for PAH and preterm PAH and developed various prediction models for PAH according to basic characteristics of mothers with MAP and PAPP-A or without them. A prediction model with basic maternal characteristics with MAP (model 2) showed the best performance and a scoring system was developed with this model. The performance of model 2 was superior to a model based on ACOG clinical risk assessment for PE [6].

The NICE [7] and ACOG [6] guidelines defined high-risk groups for PE by clinical factors in women with one or more high-risk factors or with two or more moderate risk factors and recommended aspirin prophylaxis for them. Clinical factors included in these two guidelines were different. Studies comparing performances of these two guidelines showed differences in the results by country and race. In a study on Asian subjects, the ACOG guidelines showed better performance than NICE [19]. High risk factors in the ACOG guidelines are history of PE, multifetal gestation, chronic hypertension, diabetes, renal disease, autoimmune disease. Moderate risk factors in the ACOG guidelines are nulliparity, obesity (BMI: 30 kg/m^2^), African American race, low socioeconomic status, age 35 years or older, and personal history factors (e.g., low birth weight or small for gestational age, previous adverse pregnancy outcome, more than 10-year pregnancy interval). However, maternal age and multiple pregnancies have increased in South Korea. The percentage of women aged 35 years or older was reported to be 35%, with the percentage of nulliparity exceeding 32.3% and multiple pregnancies exceeding 5.4% [20,21,22].

If the Korean society of obstetrics and gynecology follows the ACOG guidelines, it seems that over 30% of pregnant women in Korea require aspirin during pregnancy. The ACOG and U.S. preventive services task force team have suggested a high-risk group for preventive aspirin treatment when the risk of PE is over 8% [6]. In our scoring system, if the score exceeds 13 points, the risk becomes greater than 8%. Only MAP ≥ 97 mmHg before 20 weeks of gestation had a high risk (24 points with risk over 8%) as a single factor. A revised guideline by the American College of Cardiology and American Heart Association in 2017 defined high blood pressure (130–190/80–90 mmHg) as a stage 1 hypertension and suggested pharmacologic treatment in a non-pregnant status [23]. However, the ACOG continues to support a diagnosis of chronic hypertension in pregnancy when blood pressure is confirmed to be ≥140/90 mmHg [15]. There is increasing evidence supporting an association between stage 1 hypertension and the development of PE [24,25,26], as well as the benefits of treatment for mild chronic hypertension during pregnancy to reduce adverse pregnancy outcomes without impairing fetal growth [27]. In 2022, the SMFM recommended treatment with antihypertensive therapy for mild chronic hypertension in pregnancy to a goal BP of <140/90 mmHg based on recent evidence [28]. As an MAP calculated by 130/80 mmHg becomes 97 mmHg, which is correlated with the cut off value of high-risk factor in this study, those with blood pressure of 130–190/80–90 mmHg before 20 weeks of gestation might need close observation.

In this study, other risk factors including maternal age of 40 years or above, nulliparity, and multiple pregnancy showed moderate risks. In a case of a 39-year-old or 34-year-old nulliparous woman with twin gestation and initial MAP of 80 mmHg, if there are no other risk factors, the total score is 10 points, representing a risk of 5.78%. Therefore, they might not belong to the group recommended for taking aspirin. On the other hand, in the case of a 35-year-old nulliparous woman with twin gestation and diabetes, the total score is 15 points, representing a risk of 10.57%, and therefore aspirin use may be recommended. Even in a 41-year-old nulliparous woman with singleton gestation, BMI of 20 kg/m^2^, and MAP of 80 mmHg without any underling diseases, the total score is 9 points, representing a risk of 5.11%, meaning that aspirin prophylaxis is not indicated by this study model, but indicated by the ACOG criteria. Based on this study, it seems that personal health status has more importance. However, if the same woman gets pregnant with twins, the total score becomes 14 points, representing a risk of 9.39%, which may require aspirin during pregnancy.

Another considering point is the prevalence of PAH. Among East-Asian women including Chinese, Japanese, and Korean, the prevalence of hypertensive disorders in pregnancy is about 1–5%, which is much lower than those (3.3 to 15.8%) in non-Hispanic Black, African American, and Black women in US and those (0.4 to 10%) in non-Hispanic White women [29,30]. Therefore, the KSOG needs to discuss whether they should define a high-risk group for preventive aspirin treatment when the risk of PAH is over 8% or other lower cut-off values.

- Limitations and strengths of this study

This study has several limitations. Firstly, we could not extract information about family history of PE (mother or sister) or the interpregnancy interval from the EMR because it was not routinely recorded. Secondly, the MoM values of PAPP-A for the relevant gestational age were taken from routine screening records, and because the measurements of PAPP-A did not come from one central lab, there can be some bias. Thirdly, there were missing values, especially in model 3, including PAPP-A, which might have affected its performance. However, as non-invasive prenatal testing (NIPT) with high sensitivity and low false positive rate for the detection of Down syndrome was indicated mainly in high-risk pregnant women including women aged 35 years or more [31], NIPT has been widely used as an initial fetal aneuploidy screening test rather than a maternal serum screening including PAPP-A [32] in those women. Therefore, uterine artery Doppler and biochemical markers of PAPP-A and placental growth factor (PLGF) suggested by the FMF prediction model for gestational hypertension might require another cost for application of the FMF model in high-risk pregnant women [10,33]. A previous study in Asia including the FMF prediction model, FMF triple test by a combination of maternal factors, MAP, uterine artery pulsatility index, and PLGF has demonstrated a detection rate of 64.0% at 10% false-positive rate for predicting preterm PE [10]. In Korea, the PLGF test is not accepted as a first trimester screening test for predicting PE, despite the availability of the measuring soluble fms-like tyrosine kinase-1/PLGF levels after 20 weeks of gestation. This study showed sensitivities of 54.8% and 66.4% at 10% false-positive rate for predicting PAH and preterm PAH, respectively, by model 2 (a combination of maternal factors and MAP). Furthermore, it should be noted that direct comparisons with the results of other studies regarding the prediction of PE cannot be made [10,34]. In addition, although PAH in this study includes gestational hypertension, which can develop preeclampsia in the almost half of the affected women, there has been no evidence that aspirin can prevent gestational hypertension [35]. This study serves as a preliminary investigation in Korea, encompassing a comprehensive analysis of all available components related to PE.

Although model 2 suggested by this study needs future validation with uterine artery pulsatility index and PAPP-A or without them, our prediction model 2 in a test set and a restricted population showed similar results. The other strength of this study was the BMI classification [36]. The BMI classification into 5 categories for Asian women by the WHO and Korean society for the study of obesity [37,38] provided wide variations in the scores from −3 to 11 points, representing risks from 1.10% to 6.54% in this study.

Although the prevalence of PAH is relatively low in East Asian women, it has been consistently reported that PAH is associated with risks of postpartum hypertension, type 2 diabetes, hyperlipidemia, and cardiovascular diseases across racial and ethnic groups [29,30,39]. In addition, PAH has been suggested to contribute to the development of offspring cardiovascular disease and diabetes later in life [40,41]. Therefore, a prevention strategy for PAH is important not only for decreasing perinatal complications, but also for improving long term health of mothers and their offspring. A recent early prediction model study from Korea using machine learning methods, with clinical factors and blood pressure only, reported an AUC of 0.89 in the training set and 0.81 in the test set, with a sensitivity of 72.7% in the training set and 45.5% in the test set, which showed significantly better performance than a model with PLGF [42]. Although investigating important biomarkers for prediction is important, more studies about the assessment of individual risks of PAH with easily available clinical factors and blood pressure might be more important before introducing several biomarkers.

## 5. Conclusions

This preliminary study in Korea developed an early pregnancy risk scoring model for PAH according to basic characteristics of mothers and MAP. A future prospective study for validating this scoring system with biomarkers and uterine artery Doppler (or without them) might be required when discussing the cut-off risk of PAH in East Asian pregnant women.

## Figures and Tables

**Table 1:** Baseline and clinical characteristics in train sets.

	PAH (N = 1988)	Preterm PAH (N = 1085)	Control (N = 22,514)	Analysis 1 *p*-Value	Analysis 2 *p*-Value
Maternal age, years, mean ± SD	33.30 ± 4.84	33.26 ± 4.68	32.74 ± 4.42	<0.0001	<0.0001
Ethnicity				0.4778	0.3666
Korean	1935 (97.33)	1053 (97.05)	21,937 (97.44)		
Northeast Asian	20 (1.01)	15 (1.38)	217 (0.96)		
Southwest Asian	23 (1.16)	12 (1.11)	291 (1.29)		
Uncertain	10 (0.50)	5 (0.46)	69 (0.31)		
Paternal age, years, mean ± SD	35.73 ± 5.09	35.48 ± 5.06	35.37 ± 4.81	0.004	0.3477
Nulliparity, n (%)	1219 (61.32)	646 (59.54)	11,726 (52.08)	<0.0001	<0.0001
IVF, n (%)	127 (6.39)	83 (7.65)	960 (4.26)	<0.0001	<0.0001
Pre-pregnant BMI (kg/m^2^), mean ± SD	24.36 ± 5.10	24.12 ± 4.87	21.55 ± 3.49	<0.0001	<0.0001
Pre-pregnant BMI (kg/m^2^), n (%)				<0.0001	<0.0001
BMI < 25 kg/m^2^, n (%)	1237 (62.70)	695 (64.47)	19,403 (86.49)		
BMI ≥ 25 kg/m^2^, < 30 kg/m^2^, n (%)	465 (23.57)	258 (23.93)	2344 (10.45)		
BMI ≥ 30 kg/m^2^, n (%)	271 (13.74)	125 (11.60)	686 (3.06)		
Pre-pregnant smoking history, n (%)	49 (2.46)	19 (1.75)	324 (1.44)	0.0009	0.5782
Pre-pregnant drinking history, n (%)	21 (1.06)	7 (0.65)	127 (0.56)	0.0092	0.8186
Family history of hypertension, n (%)	644 (32.39)	330 (30.41)	4289 (19.05)	<0.0001	<0.0001
Family history of diabetes, n (%)	259 (13.03)	136 (12.53)	2626 (11.66)	0.0704	0.3354
History of a previous pregnancy					
Preterm birth, n (%)	195 (9.81)	130 (11.98)	1348 (5.99)	<0.0001	<0.0001
Preeclampsia n (%)	151 (7.60)	78 (7.19)	293 (1.30)	<0.0001	<0.0001
FDIU, n (%)	24 (1.21)	15 (1.38)	159 (0.71)	<0.0001	<0.0001
GDM, n (%)	41 (2.06)	21 (1.94)	378 (1.68)	<0.0001	<0.0001
FGR, n (%)	60 (3.02)	35 (3.23)	412 (1.83)	<0.0001	<0.0001
Preexisting diseases					
Chronic hypertension, n (%)	362 (18.21)	177 (16.31)	597 (2.65)	<0.0001	<0.0001
Diabetes, n (%)	60 (3.02)	30 (2.76)	211 (0.94)	<0.0001	<0.0001
Renal disease, n (%)	74 (3.72)	46 (4.24)	186 (0.83)	<0.0001	<0.0001
PCOS, n (%)	40 (2.01)	15 (1.38)	487 (2.16)	0.6564	0.0735
IGT, n (%)	14 (0.70)	8 (0.74)	81 (0.36)	0.0178	0.0795
Hyperlipidemia, n (%)	96 (4.83)	44 (4.06)	451 (2.00)	<0.0001	<0.0001
Lupus or APS, n (%)	40 (2.01)	26 (2.40)	182 (0.81)	<0.0001	<0.0001
Rheumatic arthritis, n (%)	31 (1.56)	13 (1.20)	447 (1.99)	0.1879	0.0393
Other rheumatic diseases, n (%)	24 (1.21)	14 (1.29)	126 (0.56)	0.0004	0.0029
Aplastic anemia, n (%)	13 (0.65)	9 (0.83)	80 (0.36)	0.038	0.0479
Initial MAP (mmHg) ^1^, mean ± SD	95.40 ± 14.44	100.21 ± 17.44	83.36 ± 9.73	<0.0001	<0.0001
(n = 4822 women)					
PAPP-A (MoM) ^2^, mean ± SD	1.04 ± 0.69	1.04 ± 0.71	1.19 ± 0.66	<0.0001	0.0044
(n = 4748 women)					
Obstetric outcomes in this pregnancy					
Subgroups of PAH					
gestational hypertension, n (%)	523 (26.31)	176 (16.22)			
preeclampsia, n (%)	1244 (62.57)	770 (70.97)			
superimposed preeclampsia, n (%)	199 (10.01)	120 (11.06)			
eclampsia, n (%)	22 (1.11)	19 (1.75)			
unspecified maternal hypertension, n (%)	105 (5.28)	45 (4.15)			
GDM, n (%)	262 (13.18)	112 (10.32)	1693 (7.52)	<0.0001	0.0005
Multiple pregnancy, n (%)	164 (8.25)	128 (11.80)	1228 (5.45)	<0.0001	<0.0001
Cesarean section, n (%)	1465 (73.69)	933 (85.99)	10,404 (46.21)	<0.0001	<0.0001
Gestational age at delivery (weeks),	35.57 ± 3.51	33.33 ± 3.05	37.56 ± 3.30	<0.0001	<0.0001
mean ± SD					
Delivery < 37 weeks, n (%)	1106 (55.63)	1085 (100.00)	5193 (23.07)	<0.0001	<0.0001
Delivery < 34 weeks, n (%)	484 (24.35)	476 (43.87)	2198 (9.76)	<0.0001	<0.0001
Neonatal birth weight (kg), mean ± SD	2.37 ± 0.86	1.86 ± 0.68	2.90 ± 0.71	<0.0001	<0.0001
SGA (birth weight < 10th percentile)	583 (27.09)	418 (34.46)	1967 (8.28)	<0.0001	<0.0001

^1^ MAPs before 20 weeks’ gestation were available in 4822 women; ^2^ MoM values of PAPP-A between 11- and 13-weeks’ gestation were available in 4748 women. Analysis 1, comparison between PAH and control groups; Analysis 2, comparison between preterm PAH and control groups. PAH, pregnancy-associated hypertension; IVF, in vitro fertilization; BMI, body mass index; FDIU, fetal death in utero; GDM, gestational diabetes mellitus; FGR, fetal growth restriction; PCOS, polycystic ovary syndrome; IGT, insulin glucose tolerance; APS, antiphospholipid syndrome; MAP, mean arterial pressure; PAPP-A, pregnancy-associated plasma protein-A; MoM, multiples of median; SGA, small for gestational age. Data were presented as n (%) or mean ± SD (standard deviation).

**Table 2:** Univariate and stepwise multivariate logistic regression analyses of risk factor for pregnancy-associated hypertension.

	Univariate Analysis			Multivariate Analysis		
	OR	95% CI	*p*-Value	OR	95% CI	*p*-Value
Multiple pregnancy	1.559	(1.315–1.847)	<0.0001	1.515	(1.267–1.813)	<0.0001
Age, years	1.029	(1.019–1.040)	<0.0001	1.023	(1.011–1.034)	0.0001
Nulliparity	1.765	(1.597–1.952)	<0.0001	2.017	(1.809–2.249)	<0.0001
IVF	1.532	(1.266–1.855)	<0.0001			
History of preterm birth	0.788	(0.639–0.973)	0.0265			
History of preeclampsia	8.753	(7.078–10.825)	<0.0001	3.654	(2.843–4.697)	<0.0001
History of FDIU	2.154	(1.393–3.329)	0.0006			
History of GDM	1.551	(1.114–2.159)	0.0093			
History of FGR	2.131	(1.609–2.824)	<0.0001			
Pre-pregnant smoking history	1.731	(1.277–2.346)	0.0004			
Pre-pregnant drinking history	1.882	(1.183–2.993)	0.0076	2.019	(1.222–3.334)	0.0061
Pre-pregnant BMI	1.161	(1.150–1.173)	<0.0001	1.143	(1.131–1.155)	<0.0001
Family history of hypertension	2.036	(1.843–2.249)	<0.0001	1.645	(1.476–1.834)	<0.0001
Preexisting aplastic anemia	1.846	(1.025–3.323)	0.041			
Preexisting hypertension	8.175	(7.107–9.403)	<0.0001	4.41	(3.748–5.188)	<0.0001
Preexisting hyperlipidemia	2.483	(1.983–3.111)	<0.0001			
Preexisting diabetes	3.289	(2.460–4.398)	<0.0001	1.572	(1.119–2.208)	0.0092
Preexisting renal disease	4.642	(3.532–6.102)	<0.0001	2.137	(1.542–2.961)	<0.0001
Preexisting lupus or APS	2.521	(1.785–3.561)	<0.0001	1.722	(1.154–2.571)	0.0078
Preexisting other rheumatic diseases	2.172	(1.400–3.368)	0.0005			

OR, odds ratio; CI, confidence interval; IVF, in vitro fertilization; FDIU, fetal death in utero; GDM, gestational diabetes mellitus; FGR, fetal growth restriction; BMI, body mass index; APS, antiphospholipid syndrome.

**Table 3:** Univariate and stepwise multivariate logistic regression analyses predicting relative risk for pregnancy-associated hypertension in women with MAP.

	Univariate Analysis			Multivariate Analysis		
	OR	95% CI	*p*-Value	OR	95% CI	*p*-Value
Multiple pregnancy	1.559	(1.315–1.847)	<0.0001	2.052	(1.342–3.138)	0.0009
Age, years	1.029	(1.019–1.040)	<0.0001			
Nulliparity	1.765	(1.597–1.952)	<0.0001	1.958	(1.430–2.681)	<0.0001
IVF	1.532	(1.266–1.855)	<0.0001			
History of preterm birth	0.788	(0.639–0.973)	0.0265			
History of preeclampsia	8.753	(7.078–10.825)	<0.0001	3.788	(2.017–7.111)	<0.0001
History of FDIU	2.154	(1.393–3.329)	0.0006			
History of GDM	1.551	(1.114–2.159)	0.0093			
History of FGR	2.131	(1.609–2.824)	<0.0001			
Pre-pregnant smoking history	1.731	(1.277–2.346)	0.0004			
Pre-pregnant drinking history	1.882	(1.183–2.993)	0.0076			
Pre-pregnant BMI	1.161	(1.150–1.173)	<0.0001	1.067	(1.036–1.100)	<0.0001
Family history of hypertension	2.036	(1.843–2.249)	<0.0001			
Preexisting aplastic anemia	1.846	(1.025–3.323)	0.041			
Preexisting hypertension	8.175	(7.107–9.403)	<0.0001	3.498	(2.420–5.056)	<0.0001
Preexisting hyperlipidemia	2.483	(1.983–3.111)	<0.0001			
Preexisting diabetes	3.289	(2.460–4.398)	<0.0001	2	(1.096–3.648)	0.0238
Preexisting renal disease	4.642	(3.532–6.102)	<0.0001	2.119	(1.108–4.050)	0.0231
Preexisting lupus or APS	2.521	(1.785–3.561)	<0.0001	3.624	(1.901–6.908)	<0.0001
Preexisting other rheumatic diseases	2.172	(1.400–3.368)	0.0005			
MAP	1.09	(1.079–1.102)	<0.0001	1.066	(1.054–1.079)	<0.0001

MAP, mean arterial pressure; OR, odds ratio; CI, confidence interval; IVF, in vitro fertilization; FDIU, fetal death in utero; GDM, gestational diabetes mellitus; FGR, fetal growth restriction; BMI, body mass index; APS, antiphospholipid syndrome.

**Table 4:** Univariate and stepwise multivariate logistic regression analyses predicting relative risk for preterm pregnancy-associated hypertension.

	Univariate Analysis			Multivariate Analysis		
	OR	95% CI	*p*-Value	OR	95% CI	*p*-Value
Multiple pregnancy	1.598	(1.342–1.903)	<0.0001	2.328	(1.901–2.851)	<0.0001
Age, years	1.023	(1.012–1.034)	<0.0001	1.017	(1.002–1.032)	0.0279
Nulliparity	1.696	(1.531–1.880)	<0.0001	1.749	(1.519–2.013)	<0.0001
IVF	1.363	(1.105–1.680)	0.0038			
History of preterm birth	0.741	(0.598–0.918)	0.0062			
History of preeclampsia	8.901	(7.071–11.204)	<0.0001	3.275	(2.385–4.498)	<0.0001
History of FDIU	1.75	(1.037–2.955)	0.0361			
History of GDM	1.716	(1.221–2.411)	0.0019			
History of FGR	1.97	(1.460–2.657)	<0.0001			
Pre-pregnant smoking history	1.968	(1.463–2.649)	<0.0001			
Pre-pregnant drinking history	2.06	(1.307–3.246)	0.0018			
Pre-pregnant BMI	1.166	(1.154–1.178)	<0.0001	1.137	(1.122–1.153)	<0.0001
Family history of hypertension	1.977	(1.783–2.192)	<0.0001	1.536	(1.334–1.769)	<0.0001
Preexisting aplastic anemia	1.372	(0.712–2.644)	0.3448			
Preexisting hypertension	7.835	(6.767–9.073)	<0.0001	3.937	(3.186–4.865)	<0.0001
Preexisting hyperlipidemia	2.498	(1.974–3.161)	<0.0001			
Preexisting diabetes	3.429	(2.552–4.606)	<0.0001	1.625	(1.049–2.518)	0.0298
Preexisting renal disease	4.021	(2.984–5.418)	<0.0001	2.744	(1.855–4.058)	<0.0001
Preexisting lupus or APS	2.03	(1.261–3.268)	0.0036	2.108	(1.311–3.389)	0.0021
Preexisting other rheumatic diseases	1.148	(0.555–2.376)	0.709			

OR, odds ratio; CI, confidence interval; IVF, in vitro fertilization; FDIU, fetal death in utero; GDM, gestational diabetes mellitus; FGR, fetal growth restriction; BMI, body mass index; APS, antiphospholipid syndrome.

**Table 5:** Univariate and stepwise multivariate logistic regression analyses predicting relative risk for preterm pregnancy-associated hypertension in women with MAP.

	Univariate Analysis			Multivariate Analysis		
	OR	95% CI	*p*-Value	OR	95% CI	*p*-Value
Multiple pregnancy	2.376	(1.958–2.883)	<0.0001	3.381	(1.950–5.860)	<0.0001
Age, years	1.028	(1.014–1.042)	0.0001			
Nulliparity	1.597	(1.400–1.821)	<0.0001			
IVF	1.899	(1.504–2.398)	<0.0001			
History of preterm birth	0.639	(0.496–0.824)	0.0006			
History of preeclampsia	7.975	(6.078–10.464)	<0.0001			
History of FDIU	2.346	(1.370–4.017)	0.0019			
History of GDM	1.504	(0.958–2.362)	0.0761			
History of FGR	2.118	(1.480–3.033)	<0.0001			
Pre-pregnant smoking history	1.265	(0.793–2.017)	0.3246			
Pre-pregnant drinking history	1.276	(0.594–2.744)	0.5321			
Pre-pregnant BMI	1.15	(1.135–1.164)	<0.0001	1.079	(1.038–1.122)	0.0001
Family history of hypertension	1.827	(1.599–2.088)	<0.0001			
Preexisting aplastic anemia	2.085	(1.048–4.148)	0.0363			
Preexisting hypertension	7.194	(6.006–8.616)	<0.0001	3.678	(2.250–6.014)	<0.0001
Preexisting hyperlipidemia	2.059	(1.502–2.823)	<0.0001			
Preexisting diabetes	3.205	(2.172–4.729)	<0.0001	2.846	(1.336–6.062)	0.0067
Preexisting renal disease	5.924	(4.250–8.259)	<0.0001	3.91	(1.834–8.336)	0.0004
Preexisting lupus or APS	3.227	(2.126–4.898)	<0.0001	6.553	(2.898–14.818)	<0.0001
Preexisting other rheumatic diseases	2.27	(1.304–3.954)	0.0038			
MAP	1.111	(1.096–1.127)	<0.0001	1.083	(1.067–1.100)	<0.0001

MAP, mean arterial pressure; OR, odds ratio; CI, confidence interval; IVF, in vitro fertilization; FDIU, fetal death in utero; GDM, gestational diabetes mellitus; FGR, fetal growth restriction; BMI, body mass index; APS, antiphospholipid syndrome.

**Table 6:** Performance of scoring models for prediction of PAH and preterm PAH in total and restricted population.

	N	AIC	AUC	95% CI of AUC	Sensitivity for 10% FPR	FPR 95% CI
Prediction of PAH (total population, N = 24,502)						
Maternal factors by ACOG	24,406	12,850.122	0.665	(0.6522–0.6779)	31	(28.98–33.06)
Maternal factors by model 1	24,406	12,015.507	0.7504	(0.7386–0.7621)	41	(38.78–43.12)
Maternal factors by model 2	4817	1762.618	0.8227	(0.7963–0.8490)	54.8	(49.09–60.50)
Maternal factors by model 3	2253	782.326	0.8313	(0.7938–0.8688)	53.8	(45.28–62.42)
Prediction of PAH_R (restricted population ^1^, N = 24,036)						
Maternal factors by ACOG	23,930	12,231.051	0.6582	(0.6450–0.6715)	29.4	(27.31–31.47)
Maternal factors by model 1	23,930	11,419.799	0.748	(0.7358–0.7603)	41.5	(39.24–43.73)
Maternal factors by model 2	4581	1446.519	0.8219	(0.7915–0.8522)	52.2	(45.73–58.58)
Maternal factors by model 3	4581	1447.89	0.8223	(0.7921–0.8524)	50.4	(44.00–56.86)
Prediction of preterm PAH (total population, N = 23,599)						
Maternal factors by ACOG	23,502	8287.182	0.6534	(0.6357–0.6711)	32.4	(29.58–35.17)
Maternal factors by model 1	23,502	7859.837	0.7449	(0.7290–0.7607)	38.9	(35.96–41.78)
Maternal factors by model 2	4648	936.453	0.8764	(0.8444–0.9084)	66.4	(58.78–74.10)
Maternal factors by model 3	2227	398.474	0.859	(0.8038–0.9141)	60.3	(47.76–72.93)
Prediction of preterm PAH_R (restricted population ^1^, N = 23,193)						
Maternal factors by ACOG	23,100	7846.415	0.6577	(0.6399–0.6755)	30.9	28.03–33.74
Maternal factors by model 1	23,100	7410.101	0.7513	(0.7351–0.7675)	41.5	38.47–44.55
Maternal factors by model 2	4436	754.51	0.7851	(0.8385–0.9116)	65.5	56.57–74.34
Maternal factors by model 3	2129	330.176	0.8397	(0.7738–0.9056)	58.1	43.39–72.88

^1^ Study population without aspirin treatment; model 1, multivariable regression models with clinical factors; model 2, multivariable regression models with clinical factors and MAP; model 3, multivariable regression models with clinical factors, MAP and PAPP-A. PAH, pregnancy-associated hypertension; PAH_R, Pregnancy-associated hypertension of restricted study population without aspirin treatment; pregnancy-associated hypertension; AIC, Akaike information criterion; AUC, area under the curve; CI, confidence interval; FPR, false positive rate; ACOG, The American College of Obstetrician and Gynecologists.

## Data Availability

Data is unavailable due to privacy or ethical restrictions, according to decision by the Data Review Boards of The Catholic University of Korea.

## Appendix A

**Table A1:** Baseline and clinical characteristics in train sets of restricted population.

	PAH_R (N = 1862)	Preterm PAH_R (N = 1018)	Control (N = 22,174)	Analysis 1 *p*-Value	Analysis 2 *p*-Value
Maternal age, years, mean ± SD	33.16 ± 4.81	33.19 ± 4.71	32.71 ± 4.41	<0.0001	0.0004
Ethnicity				0.1033	0.4466
Korean	1808 (97.10)	990 (97.25)	21,594 (97.38)		
Northeast Asian	25 (1.34)	12 (1.18)	220 (0.99)		
Southwest Asian	21 (1.13)	11 (1.08)	311 (1.40)		
Uncertain	8 (0.43)	5 (0.49)	49 (0.22)		
Paternal age, years, mean ± SD	35.66 ± 5.29	35.39 ± 5.04	35.32 ± 4.78	0.0226	0.7185
Nulliparity, n (%)	1140 (61.22)	622 ± 61.10)	11,622 (52.41)	<0.0001	<0.0001
IVF, n (%)	103 (5.53)	72 (7.07)	914 (4.12)	0.0037	<0.0001
Pre-pregnant BMI (kg/m^2^), mean ± SD	24.44 ± 5.12	24.22 ± 4.80	21.54 ± 3.47	<0.0001	<0.0001
Pre-pregnant BMI (kg/m^2^), n (%)				<0.0001	<0.0001
BMI < 25 kg/m^2^, n (%)	1136 (61.61)	634 (62.96)	19,107 (86.51)		
BMI ≥ 25 kg/m^2^, < 30 kg/m^2^, n (%)	451 (24.46)	256 (25.42)	2331 (10.55)		
BMI ≥ 30 kg/m^2^, n (%)	257 (13.94)	117 (11.62)	648 (2.93)		
Pre-pregnant smoking history, n (%)	52 (2.79)	20 (1.96)	319 (1.44)	<0.0001	0.2585
Pre-pregnant drinking history, n (%)	22 (1.18)	11 (1.08)	128 (0.58)	0.0061	0.0915
Family history of hypertension, n (%)	590 (31.69)	318 (31.24)	4214 (19.00)	<0.0001	<0.0001
Family history of diabetes, n (%)	218 (11.71)	123 (12.08)	2601 (11.73)	0.9773	0.4022
History of a previous pregnancy					
Preterm birth, n (%)	166 (8.92)	105 (10.31)	1267 (5.71)	<0.0001	<0.0001
Preeclampsia n (%)	126 (6.77)	69 (6.78)	245 (1.10)	<0.0001	<0.0001
FDIU, n (%)	16 (0.86)	13 (1.28)	135 (0.61)	<0.0001	<0.0001
GDM, n (%)	39 (2.09)	19 (1.87)	340 (1.53)	<0.0001	<0.0001
FGR, n (%)	52 (2.79)	27 (2.65)	400 (1.80)	<0.0001	<0.0001
Preexisting diseases					
Chronic hypertension, n (%)	318 (17.08)	158 (15.52)	568 (2.56)	<0.0001	<0.0001
Diabetes, n (%)	58 (3.11)	27 (2.65)	206 (0.93)	<0.0001	<0.0001
Renal disease, n (%)	59 (3.17)	41 (4.03)	179 (0.81)	<0.0001	<0.0001
PCOS, n (%)	33 (1.77)	17 (1.67)	499 (2.25)	0.178	0.3324
IGT, n (%)	12 (0.64)	9 (0.88)	80 (0.36)	0.0569	0.0077
Hyperlipidemia, n (%)	87 (4.67)	37 (3.63)	427 (1.93)	<0.0001	0.0003
Lupus or APS, n (%)	20 (1.07)	12 (1.18)	118 (0.53)	0.0029	0.0143
Rheumatic arthritis, n (%)	24 (1.29)	13 (1.28)	440 (1.98)	0.0362	0.1147
Other rheumatic diseases, n (%)	8 (0.43)	2 (0.20)	83 (0.37)	0.7088	0.4425
Aplastic anemia, n (%)	10 (0.54)	5 (0.49)	87 (0.39)	0.3442	0.8048
Initial MAP (mmHg) ^1^, mean ± SD	96.70 ± 15.38	98.34 ± 16.59	83.17 ± 9.63	<0.0001	<0.0001
(n = 4586 women)					
PAPP-A (MoM) ^2^, mean ± SD	1.12 ± 0.84	1.05 ± 0.79	1.21 ± 0.67	0.0019	0.007
(n = 4593 women)					
Obstetric outcomes in this pregnancy					
Subgroups of PAH					
gestational hypertension, n (%)	501 (26.91)	184 (18.07)			
preeclampsia, n (%)	1073 (57.63)	677 (66.50)			
superimposed preeclampsia, n (%)	173 (9.29)	108 (10.61)			
eclampsia, n (%)	25 (1.34)	21 (2.06)			
unspecified maternal hypertension, n (%)	90 (4.83)	28 (2.75)			
GDM, n (%)	236 (12.67)	110 (10.81)	1656 (7.47)	<0.0001	<0.0001
Multiple pregnancy, n (%)	155 (8.32)	118 (11.59)	1192 (5.38)	<0.0001	<0.0001
Cesarean section, n (%)	1388 (74.54)	877 (86.15)	10,208 (46.04)	<0.0001	<0.0001
Gestational age at delivery (weeks),	35.56 ± 3.52	33.41 ± 2.92	37.54 ± 3.32	<0.0001	<0.0001
mean ± SD					
Delivery < 37 weeks, n (%)	1030 (55.32)	1018 (100.00)	5145 (23.20)	<0.0001	<0.0001
Delivery < 34 weeks, n (%)	465 (24.97)	431 (42.34)	2204 (9.94)	<0.0001	<0.0001
Neonatal birth weight (kg), mean ± SD	2.36 ± 0.86	1.89 ± 0.68	2.90 ± 0.71	<0.0001	<0.0001
SGA (birth weight < 10th percentile)	570 (28.26)	379 (33.36)	1884 (8.06)	<0.0001	<0.0001

^1^ MAPs before 20 weeks’ gestation were available in 4586 women; ^2^ MoM values of PAPP-A between 11- and 13-weeks’ gestation were available in 4593 women. Analysis 1, comparison between PAH_R, and control groups; Analysis 2, comparison between preterm PAH_R and control groups. PAH_R, Pregnancy-associated hypertension of restricted study population without aspirin treatment; pregnancy-associated hypertension; IVF, in vitro fertilization; BMI, body mass index; FDIU, fetal death in utero; GDM, gestational diabetes mellitus; FGR, fetal growth restriction; PCOS, polycystic ovary syndrome; IGT, insulin glucose tolerance; APS, antiphospholipid syndrome; MAP, mean arterial pressure; PAPP-A, pregnancy-associated plasma protein-A; MoM, multiples of median; SGA, small for gestational age. Data were presented as n (%) or mean ± SD (standard deviation).

**Table A2:** Univariate and stepwise multivariate logistic regression analyses predicting relative risk for pregnancy-associated hypertension in women with MAP and PAPP-A.

	Univariate Analysis			Multivariate Analysis		
	OR	95% CI	*p*-Value	OR	95% CI	*p*-Value
Multiple pregnancy	1.559	(1.315–1.847)	<0.0001	4.096	(2.013–8.335)	0.0001
Age, years	1.029	(1.019–1.040)	<0.0001			
Nulliparity	1.458	(1.327–1.602)	<0.0001			
IVF	1.532	(1.266–1.855)	<0.0001			
History of Preterm birth	0.788	(0.639–0.973)	0.0265			
History of Preeclampsia	8.753	(7.078–10.825)	<0.0001	4.488	(1.864–10.806)	0.0008
History of FDIU	2.154	(1.393–3.329)	0.0006			
History of GDM	1.551	(1.114–2.159)	0.0093			
History of FGR	2.131	(1.609–2.824)	<0.0001			
Pre-pregnant smoking history	1.731	(1.277–2.346)	0.0004			
Pre-pregnant drinking history	1.882	(1.183–2.993)	0.0076			
Pre-pregnant BMI	1.161	(1.150–1.173)	<0.0001	1.089	(1.038–1.142)	0.0005
Family history of hypertension	2.036	(1.843–2.249)	<0.0001			
Preexisting aplastic anemia	1.846	(1.025–3.323)	0.041			
Preexisting hypertension	8.175	(7.107–9.403)	<0.0001	3.522	(2.119–5.853)	<0.0001
Preexisting hyperlipidemia	2.483	(1.983–3.111)	<0.0001			
Preexisting diabetes	3.289	(2.460–4.398)	<0.0001			
Preexisting renal disease	4.642	(3.532–6.102)	<0.0001	2.645	(1.138–6.147)	0.0238
Preexisting lupus or APS	2.521	(1.785–3.561)	<0.0001	3.995	(1.703–9.375)	0.0015
Preexisting other rheumatic diseases	2.172	(1.400–3.368)	0.0005			
MAP	1.09	(1.079–1.102)	<0.0001	1.055	(1.036–1.075)	<0.0001
PAPP-A	0.418	(0.290–0.603)	<0.0001	0.473	(0.265–0.844)	0.0113

MAP, mean arterial pressure; PAPP-A, pregnancy-associated plasma protein-A; OR, odds ratio; CI, confidence interval; FDIU, fetal death in utero; GDM, gestational diabetes mellitus; FGR, fetal growth restriction; BMI, body mass index; IVF, in vitro fertilization; APS, antiphospholipid syndrome; MoM, multiples of median.

**Table A3:** Univariate and stepwise multivariate logistic regression analyses predicting relative risk for preterm pregnancy-associated hypertension in women with MAP and PAPP-A.

	Univariate Analysis			Multivariate Analysis		
	OR	95% CI	*p*-Value	OR	95% CI	*p*-Value
Multiple pregnancy	2.376	(1.958–2.883)	<0.0001	6.267	(2.369–16.578)	0.0002
Age, years	1.028	(1.014–1.042)	0.0001			
Nulliparity	1.348	(1.191–1.527)	<0.0001			
IVF	1.899	(1.504–2.398)	<0.0001			
History of preterm birth	0.639	(0.496–0.824)	0.0006			
History of preeclampsia	7.975	(6.078–10.464)	<0.0001			
History of FDIU	2.346	(1.370–4.017)	0.0019			
History of GDM	1.504	(0.958–2.362)	0.0761			
History of FGR	2.118	(1.480–3.033)	<0.0001			
Pre-pregnant smoking history	1.265	(0.793–2.017)	0.3246			
Pre-pregnant drinking history	1.276	(0.594–2.744)	0.5321			
Pre-pregnant BMI	1.15	(1.135–1.164)	<0.0001			
Family history of hypertension	1.827	(1.599–2.088)	<0.0001			
Preexisting aplastic anemia	2.085	(1.048–4.148)	0.0363			
Preexisting hypertension	7.194	(6.006–8.616)	<0.0001	3.427	(1.658–7.082)	0.0009
Preexisting hyperlipidemia	2.059	(1.502–2.823)	<0.0001			
Preexisting diabetes	3.205	(2.172–4.729)	<0.0001	6.145	(2.026–18.633)	0.0013
Preexisting renal disease	5.924	(4.250–8.259)	<0.0001	5.299	(1.938–14.489)	0.0012
Preexisting lupus or APS	3.227	(2.126–4.898)	<0.0001	8.227	(2.994–22.605)	<0.0001
Preexisting other rheumatic diseases	2.27	(1.304–3.954)	0.0038			
MAP	1.111	(1.096–1.127)	<0.0001	1.081	(1.056–1.105)	<0.0001
PAPP-A	0.366	(0.204–0.658)	0.0008	0.417	(0.181–0.959)	0.0395

MAP, mean arterial pressure; PAPP-A, pregnancy-associated plasma protein-A; OR, odds ratio; CI, confidence interval; IVF, in vitro fertilization; FDIU, fetal death in utero; GDM, gestational diabetes mellitus; FGR, fetal growth restriction; BMI, body mass index; APS, antiphospholipid syndrome; MoM, multiples of median.
